# Structural and surface characterizations of 2D β-In_2_Se_3_/3D β-Ga_2_O_3_ heterostructures grown on c-Sapphire substrates by molecular beam epitaxy

**DOI:** 10.1038/s41598-024-55830-y

**Published:** 2024-03-01

**Authors:** Umeshwar Reddy Nallasani, Ssu-Kuan Wu, Nhu Quynh Diep, Yen-Yu Lin, Hua-Chiang Wen, Wu-Ching Chou, Chin-Hau Chia

**Affiliations:** 1 Department of Electrophysics, College of Science, National Yang Ming Chiao Tung University, 1001 University Road, Hsinchu, 300093 Taiwan, ROC; 2 Department of Applied Physics, National University of Kaohsiung, 700 Kaohsiung University Road, Kaohsiung, 81148 Taiwan, ROC

**Keywords:** 2D layered materials, In_2_Se_3_, Ga_2_O_3_, Mixed-dimensional heterostructure, Molecular beam epitaxy, RHEED, Two-dimensional materials, Two-dimensional materials

## Abstract

Integrating two-dimensional (2D) layered materials with wide bandgap β-Ga_2_O_3_ has unveiled impressive opportunities for exploring novel physics and device concepts. This study presents the epitaxial growth of 2D β-In_2_Se_3_/3D β-Ga_2_O_3_ heterostructures on c-Sapphire substrates by plasma-assisted molecular beam epitaxy. Firstly, we employed a temperature-dependent two-step growth process to deposit Ga_2_O_3_ and obtained a phase-pure $(\overline{2 }01)$ β-Ga_2_O_3_ film on c-Sapphire. Interestingly, the in-situ reflective high-energy electron diffraction (RHEED) patterns observed from this heterostructure revealed the in-plane ‘b’ lattice constant of β-Ga_2_O_3_ ~ 3.038Å. In the next stage, for the first time, 2D In_2_Se_3_ layers were epitaxially realized on 3D β-Ga_2_O_3_ under varying substrate temperatures (T_sub_) and Se/In flux ratios (R_VI/III_). The deposited layers exhibited (00*l*) oriented β-In_2_Se_3_ on $(\overline{2 }01)$ β-Ga_2_O_3_/c-Sapphire with the epitaxial relationship of $[11\overline{2 }0]$ β-In_2_Se_3_ || [010] β-Ga_2_O_3_ and $[10\overline{1 }0]$ β-In_2_Se_3_ || [102] β-Ga_2_O_3_ as observed from the RHEED patterns. Also, the in-plane ‘a’ lattice constant of β-In_2_Se_3_ was determined to be ~ 4.027Å. The single-phase β-In_2_Se_3_ layers with improved structural and surface quality were achieved at a T_sub_ ~ 280 °C and R_VI/III_ ~ 18. The microstructural and detailed elemental analysis further confirmed the epitaxy of 2D layered β-In_2_Se_3_ on 3D β-Ga_2_O_3_, a consequence of the quasi-van der Waals epitaxy. Furthermore, the β-Ga_2_O_3_ with an optical bandgap (E_g_) of ~ 5.04 eV (deep ultraviolet) when integrated with 2D β-In_2_Se_3_, E_g_ ~ 1.43eV (near infra-red) can reveal potential applications in the optoelectronic field.

## Introduction

Since the advent of two-dimensional (2D) layered van der Waals (vdWs) materials, Indium Selenide (In_x_Se_y_), one of the prominent candidates in this family, has been widely explored in the scientific community to investigate its novel and impeccable properties^1–8^. It belongs to a complex system that crystallizes into different stoichiometric ratios (stacking configurations), e.g., InSe (β, γ), In_2_Se_3_ (α, β, γ and δ), In_3_Se_4,_ etc., under various deposition conditions and growth techniques^4,7,8^. Among these, the β-In_2_Se_3_ with its rhombohedral crystal structure has a primitive unit cell (a = b = 4.00 Å, and c = 28.33 Å) comprising three monolayers that are stacked vertically and repeatedly by weak vdW forces, with an in-plane covalently bonded atomic sequence of “Se − In − Se − In − Se”^2^. The β-In_2_Se_3_ is renowned for its exceptional chemical stability and remarkable optical activity at room temperature (RT) and further exhibits strong 2D quantum confinement effects with its absorption edge in the near infra-red (IR) spectral range (~ 1.43 eV)^1,2^. In addition, recent studies demonstrated that the phase-engineering of In_2_Se_3_ from α to β through thermal annealing has resulted in ultrahigh responsivity and detectivity of 8.8 × 10^4^ A/W and 2.9 × 10^13^ Jones, respectively^6^.

On the other hand, recently, there has been significant research interest in exploring the integration of 2D layered materials with wide bandgap (WB) semiconductors, particularly Gallium Oxide (Ga_2_O_3_)^9–13^. Being a fourth-generation semiconducting material, Ga_2_O_3_, one of the group-III metal sesquioxide exhibits various polymorphs: α, β, γ, δ, and ε^14^. Among which the monoclinic β-phase (a = 12.23 Å, b = 3.04 Å, c = 5.80 Å, and β = 103.71°) with its direct bandgap (E_g_) ~ 4.9 eV is considered to be the thermodynamically stable structure^14,15^. Due to its ultrawide E_g_, high breakdown electric field of ~ 8 MV/cm, and robust chemical/thermal stability, it has exhibited tremendous progress in high-power electronics and deep ultraviolet (UV) optoelectronic devices^16–18^. Integrating this material with 2D layered materials can unveil novel opportunities in device physics. For instance, Wang et al. demonstrated a solar-blind photodetector with p-GaSe/n-Ga_2_O_3_ vdWs heterostructure that showed a high responsivity of 51.9 A/W and a pronounced specific detectivity up to 10^14^ Jones, resulting from the efficient separation of charge carriers across the pn junctions^11^. An ambipolar p-TMD (p-MoTe_2_ or p-WSe_2_)/n-Ga_2_O_3_ junction field effect transistor (JFET) was reported by Choi et al., with two different types of channels in a single device architecture with their respective charge carriers^12^. Despite the challenge in realizing the enhancement mode (e-mode) operation of the Ga_2_O_3_ device due to lack of p-type doping, Yang et al. fabricated a β-Ga_2_O_3_ FET with ferroelectric α-In_2_Se_3_ wrapped-gate that changed from depletion- to e-mode operation by effectively controlling the threshold voltage^13^. These findings collectively highlight the significance and potential of 2D material/β-Ga_2_O_3_ heterostructures for future device applications.

Nevertheless, the integration of these 2D materials with WB-Ga_2_O_3_ from the previous works was constrained to the ex-situ techniques, particularly by exfoliation or transfer methods of either the 2D vdW layers or the underlying Ga_2_O_3_ layers from the bulk substrates. Although the results are encouraging, such methods offer limited control over film thickness, may be prone to contamination and defects, and, most importantly, accessible with reduced scalability, therefore limiting their usage in large-area applications. Owing to these challenges, the utilization of molecular beam epitaxy (MBE) emerges as a proven growth technique to fabricate these heterostructures in situ with its ultra-high vacuum (UHV) conditions, high pure elements, thickness controllability and further yielding single crystalline materials with reduced defects.

For the first time in this study, the mixed-dimensional 2D β-In_2_Se_3_/3D β-Ga_2_O_3_ heterostructures were realized in situ using plasma-assisted molecular beam epitaxy (PA-MBE) on c-Sapphire. To achieve high-quality heteroepitaxial films, careful optimization of the initial β-Ga_2_O_3_ growth process is essential. A strategic approach involves the introduction of a low-temperature (LT) buffer (nucleation) layer, which proves effective in two key aspects. Firstly, the LT buffer layer serves as a sacrificial template by incorporating and localizing threading dislocations (TDs) that arise due to the lattice mismatch concerning the substrate^19,20^. Secondly, it provides a homo-surface, circumventing lattice constraints^21^ and facilitating a smoother transition for high-temperature (HT) film growth. Consequently, we used a two-stepped β-Ga_2_O_3_ film grown under LT and HT conditions, commonly used for the heteroepitaxy on a Sapphire substrate, to improve the crystal quality effectively^19,21^. Amidst the daunting challenge of the inherent and uneven surface of 3D Ga_2_O_3_, we successfully achieved the epitaxy of 2D In_2_Se_3_, thanks to our vigilant in-situ reflective high-energy electron diffraction (RHEED) tool for providing the information about the structural changes, in-plane lattice constants, and epitaxial relationships of the grown films. Besides the rich phases of In_2_Se_3,_ we achieved the dominant phase 2D β-In_2_Se_3_ on 3D β-Ga_2_O_3,_ which was confirmed by X-ray Diffraction (XRD) and Raman Spectroscopy. Furthermore, the surface morphological changes of the grown layers were studied carefully using Atomic Force Microscope (AFM) measurements. The microstructural and detailed elemental analysis across the heterostructures grown on c-Sapphire was thoroughly investigated by (Scanning) Transmission Electron Microscopy-(S)TEM measurements. The results presented in this study establish a fundamental understanding of the epitaxy of 2D In_2_Se_3_/3D Ga_2_O_3_ heterostructures, which is crucial for its commercialization in large-area applications.

## Results and discussion

**Figure 1:**
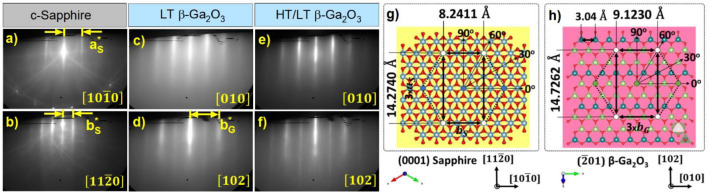
In-situ RHEED patterns of (**a**,**b**) c-Sapphire substrate (before growth), (**c**,**d**) LT β-Ga_2_O_3,_ and (**e**,**f**) HT/LT β-Ga_2_O_3_ films at ~ 5 min of their respective growths. The in-plane crystallographic views of (**g**) (0001) Sapphire and (**h**) $(\overline{2 }01)$ β-Ga_2_O_3_ along the growth direction were visualized using the ball and stick model by VESTA Software^42^.

Figure 1a,b shows the in-situ RHEED patterns of the c-Sapphire substrate (before growth). Soon after the growth of LT-Ga_2_O_3_ film (substrate temperature, T_sub_ ~ 450 °C), the transition in the RHEED patterns occurred along both the azimuthal directions (repeated for every 60° rotation), as shown in Fig. 1c,d, indicating the change in crystal structure from rhombohedral (α) c-Sapphire to monoclinic (β) Ga_2_O_3_. The in-plane epitaxial relationship observed from RHEED patterns revealed that the Ga_2_O_3_ was aligned along [010] β-Ga_2_O_3_ || $[10\overline{1 }0]$ c-Sapphire and [102] β-Ga_2_O_3_ || $[11\overline{2 }0]$ c-Sapphire, and the respective growth directional views were showed in Fig. S1. This preferential alignment of β-Ga_2_O_3_ is attributed to its similar oxygen atomic arrangement compared to c-Sapphire, and the effort to minimize lattice strain to establish a coherent epitaxial relationship between them. This is attributed to the fact that the lattice points within the growth directional planes, (0001) Sapphire and $(\overline{2 }01)$ β-Ga_2_O_3,_ maintain closer repeated interatomic distances (rectangle with white circled corners), as shown in Fig. 1g,h. Further exerting an in-plane lattice mismatch of -3.2% and -10.7% (minus indicates compressively strained β-Ga_2_O_3_) in their respective directions. This mismatch might arise due to the slight distortion of regular hexagon redistribution of oxygen atoms with a single O–O distance: 4.76 Å in (0001) Sapphire to two O–O distances: 4.96 Å and 5.15 Å in $(\overline{2 }01)$ β-Ga_2_O_3_^15^, owing to the change in ionic radii of the Al^3+^ (0.54 Å) to Ga^3+^ (0.62 Å)^22^. Moreover, this initially grown LT-Ga_2_O_3_ can serve as a nucleation film between the c-Sapphire and the HT-Ga_2_O_3_ (T_sub_ ~ 700 °C) film by minimizing this lattice and in-plane thermal expansion (α) mismatches between (0001)-Sapphire (α_s_ ~ 5 × 10^–6^ K^−1^)^23^ and $(\overline{2 }01)$ β-Ga_2_O_3_ (α_g_ ~ 7.8 × 10^–6^ K^−1^)^24^. Corroborating this, the evolution of RHEED patterns from the LT β-Ga_2_O_3_ film grown on c-Sapphire exhibited a significant improvement in the crystalline quality from starting to finishing growth at T_sub_ ~ 450 °C, as shown in Fig S2. Furthermore, Fig. 1e,f shows sharper and streakier RHEED patterns observed from the HT-grown Ga_2_O_3_ film. This could result from the lattice mismatch compensation and uniform nucleation in the LT-Ga_2_O_3_ film, providing a decent surface for the homoepitaxy at HT. More detailed information on the temporal evolution of RHEED patterns from the two-stepped (HT/LT) β-Ga_2_O_3_ film indicating the improvement in crystalline quality is discussed in the supplementary information.

**Figure 2:**
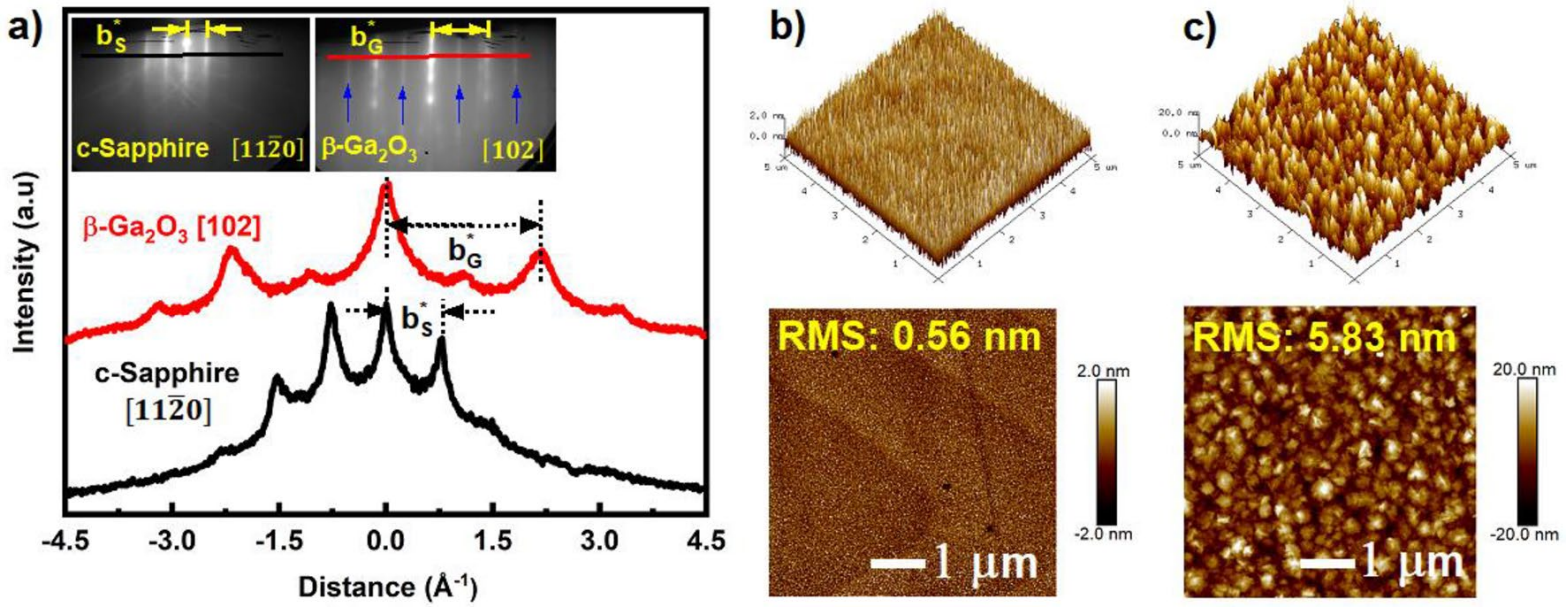
(**a**) RHEED intensity profiles of $[11\overline{2 }0]$ c-Sapphire and [102] β-Ga_2_O_3_ (HT/LT) after growth, with the in-plane ‘b’ lattice constant evaluated to be ~ 3.038 Å. The inset shows the corresponding patterns, and the profiles are extracted from respectively. 5 × 5 µm^2^ AFM scans of (**b**) LT-Ga_2_O_3_ and (**c**) HT/LT-Ga_2_O_3_ exhibiting a smaller and larger granular morphology, respectively.

Notably, the information about the 'b' lattice constant of the β-Ga_2_O_3_ crystal structure can be obtained from the RHEED pattern along [102] β-Ga_2_O_3_. In this regard, we extracted the RHEED intensity profiles along respective directions, as shown in Fig. 2a. The reciprocal lattice spacing of ${b}_{{G}_{[102]}}^{*}$= 2.068 Å^−1^ extracted from known lattice distance of c-Sapphire along ${b}_{{S}_{[11\overline{2 }0]}}$= 8.241 Å (${\sqrt{3}a}_{S}$)^25^ in real space, has yielded the ‘b’ lattice constant of β-Ga_2_O_3_, ${b}_{{G}_{\left[102\right]}}$= 2π/${b}_{{G}_{[102]}}^{*}$ Å ~ 3.038 Å, which matches well with the theoretical value^15,24^, which could indicate the fully relaxed β-Ga_2_O_3_ film grown on c-Sapphire. This was determined quantitatively by the translation of streak spacing in the reciprocal lattice by the number of pixels achieved from the RHEED patterns^26^. The additional diffraction streaks observed in this direction (indicated by blue arrows) also maintained a similar streak spacing. This coexistence of patterns along [102] β-Ga_2_O_3_ might arise from the octahedral and tetrahedral planes of Ga atoms within the $(\overline{2 }01)$ β-Ga_2_O_3_; however, further understanding is required to confirm its origin. The surface morphology of the as-grown LT-Ga_2_O_3_ and two-stepped Ga_2_O_3_ films grown on c-Sapphire is shown in Fig. 2b,c from the AFM scans. At LT, the surface of the film exhibited small granular morphology with dense grain boundaries owing to a root mean square (RMS) of ~ 0.56 nm. This is because the adatoms at LT will not have sufficient energy to transfer and nucleate with adjacent atoms on the surface, thus resulting in high nucleation sites, as seen in Fig. 2b. On the other hand, this decreased mobility of surface species can promote uniform dispersion of nuclei that can effectively cover the substrate^19^ and provide a homo-surface for the HT film growth. At HT conditions, Fig. 2c, the surface of the film was covered with large grains with reduced grain boundaries and exhibited a rougher surface (RMS ~ 5.83 nm). This could be ascribed to the greater likelihood of an adatom encountering an existing island formed during the ripening stage and promoting further growth primarily due to an increased adatom diffusion coefficient at HT.

**Figure 3:**
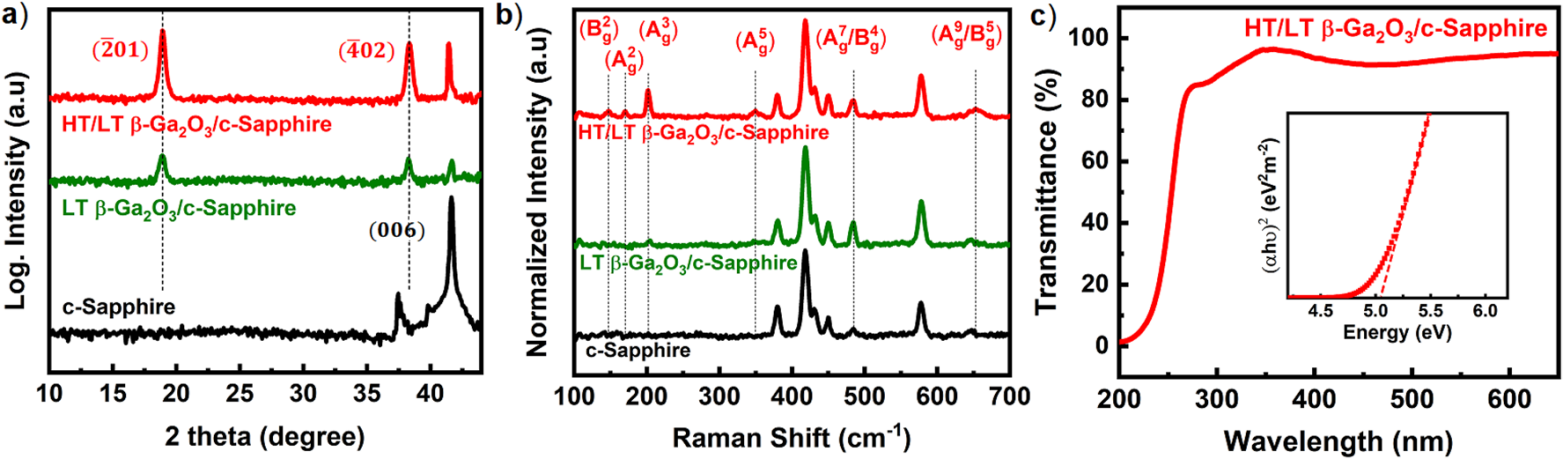
(**a**) XRD 2θ-scans and (**b**) Raman Spectra of LT β-Ga_2_O_3_ (green) and HT/LT β-Ga_2_O_3_ (red) grown on a c-Sapphire substrate (black). (**c**) Transmittance spectrum of HT/LT β-Ga_2_O_3_. Inset shows the plot of (αhν)^2^ versus photon energy, where α and hν represent the absorption coefficient and photon energy, respectively. The optical bandgap of ~ 5.04 eV was estimated by extrapolating α to 0.

Figure 3a shows XRD 2θ-scans of the Ga_2_O_3_ films grown on c-Sapphire. The LT nucleation film exhibited distinct diffraction peaks at ~ 18.9° and ~ 38.3°, corresponding to the $(\overline{2 }01)$ and $(\overline{4 }02)$ diffraction peaks of β-Ga_2_O_3_, respectively^15,17^ (ICDD Card No. 01–082-3838). Moreover, the ratio calculated between these $(\overline{2 }01)$ and $(\overline{4 }02)$ diffraction peaks was found to be ~ 1.89 (ideal value ~ 2.2)^27^, suggesting the dominant β-Ga_2_O_3_ when grown at a low T_sub_ ~ 450 °C, in contrast to the observation of secondary phases by Oshima et al.^27^ Also, the d-spacing measured between the $(\overline{2 }01)$ planes of LT β-Ga_2_O_3_ is determined to be ~ 0.468 nm, with the thickness of film ~ 32 nm as shown by TEM images in the supplementary information, Fig. S3. Further, depositing the HT Ga_2_O_3_ layers, the $(\overline{2 }01)$ family of 2θ diffraction peaks persisted by preserving the single oriented β-phase. The employment of LT nucleation film presents a key advantage in minimizing the likelihood of defects propagating into the HT film due to lattice mismatch^19,20^. Additional information on the crystalline quality of Ga_2_O_3_ without and with LT nucleation film is shown in Fig. S4, suggesting the improved Ga_2_O_3_ film quality with incorporating LT nucleation film. The Raman spectra of the Ga_2_O_3_ films grown on c-Sapphire exhibited the phonon modes corresponding to β-phase, as shown in Fig. 3b. These peaks are segregated into three categories: the lower frequency peaks located ~ 147.3 cm^−1^(B_g_^2^), ~ 170.5 cm^−1^(A_g_^2^), and ~ 202.0 cm^−1^(A_g_^3^) are attributed to libration and translation of octahedral-tetrahedral chains, the mid-frequency peaks located ~ 350.3 cm^−1^(A_g_^5^), and ~ 483.7 cm^−1^(A_g_^7^/B_g_^4^) are attributed to deformation of GaO_6_ octahedra, and the higher frequency peak located ~ 656.7 cm^−1^(A_g_^9^/B_g_^5^) relates to the stretching and bending of GaO_4_ tetrahedra^28–30^. The more pronounced vibrational modes were observed from the two-stepped film with the FWHM of A_g_^3^ mode ~ 5.5 cm^−1^. Figure 3c shows the transmittance spectra of the HT/LT β-Ga_2_O_3_ film at RT, and the spectrum exhibited an average transmittance of ~ 94% with clear interference fringes in the visible region. The direct bandgap of a semiconductor can be determined from the UV–visible spectra by using the Tauc relation ${\left(\alpha h\nu \right)}^{2}\propto {(h\nu -E}_{g}$)^31^, where α is the absorption coefficient, hν is the incident photon energy, and E_g_ is the optical bandgap. An abrupt decrement in the wavelength was observed at the absorption edge around 250 nm, indicating the presence of an optical bandgap. The E_g_ of the β-Ga_2_O_3_ film is estimated by extrapolating the intercept of the energy axis at α = 0 with an approximate value ~ 5.04 eV (deep UV region), similar to previously reported values^30,32,33^. By virtue of the successful epitaxy of two-stepped β-Ga_2_O_3_ film on c-Sapphire, in the following crucial stage, we focused on depositing and investigating the 2D-In_2_Se_3_ films on 3D β-Ga_2_O_3_ using MBE.

**Figure 4:**
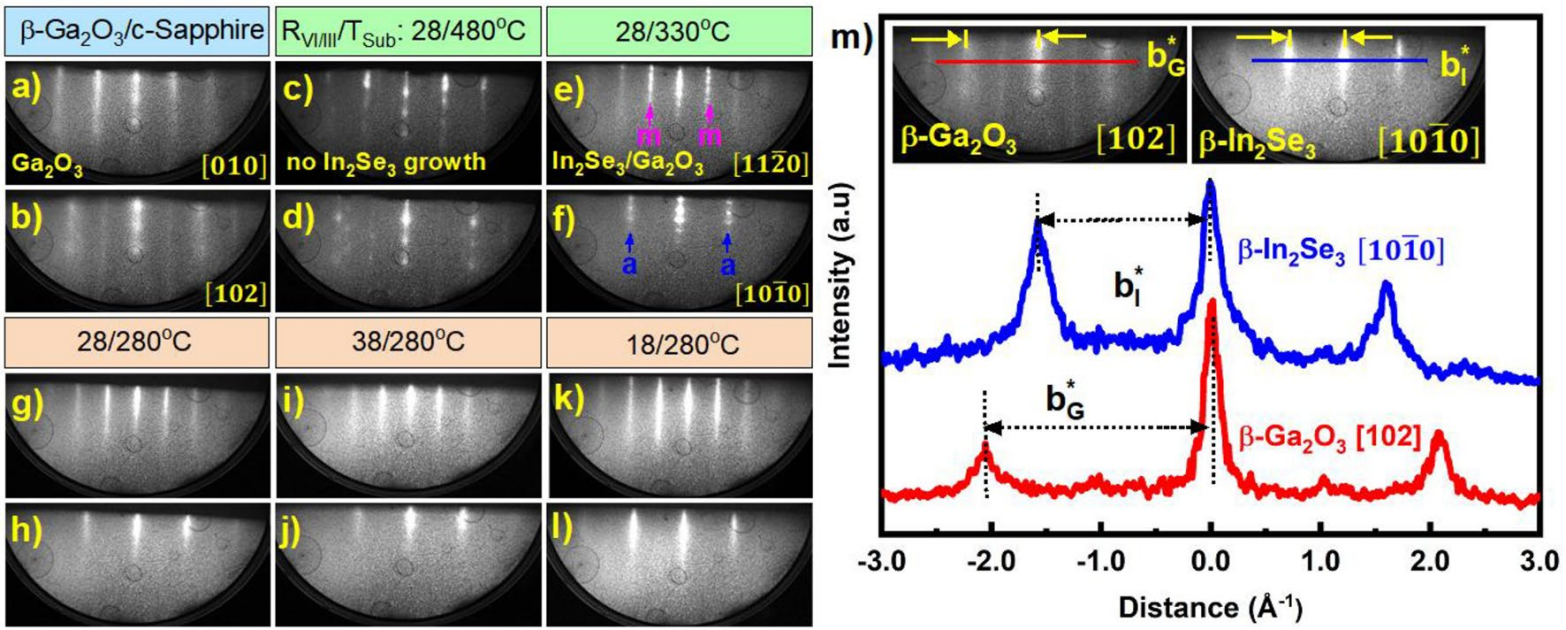
In-situ RHEED patterns of (**a**,**b**) as-grown HT/LT β-Ga_2_O_3_/c-Sapphire, with the epitaxy of In_2_Se_3_ layers grown on it under varied R_VI/III/_T_sub_ conditions, (**c**,**d**) 28/480 °C, (**e**,**f**) 28/330 °C, where m and a are denoted as the diffraction planes from hexagonal crystal symmetry and along $[11\overline{2 }0]$ and $[10\overline{1 }0]$ azimuth rotations, (**g**,**h**) 28/280 °C, (**i**,**j**) 38/280 °C and (**k**,**l**) 18/280 °C. (m) RHEED intensity profiles of [102] HT/LT β-Ga_2_O_3_ and $[10\overline{1 }0]$ β-In_2_Se_3_ (18/280 °C) after growth, with the in-plane lattice constant evaluated to be ~ 4.027 Å. The inset shows the corresponding patterns, and the profiles are extracted from respectively.

**Figure 5:**
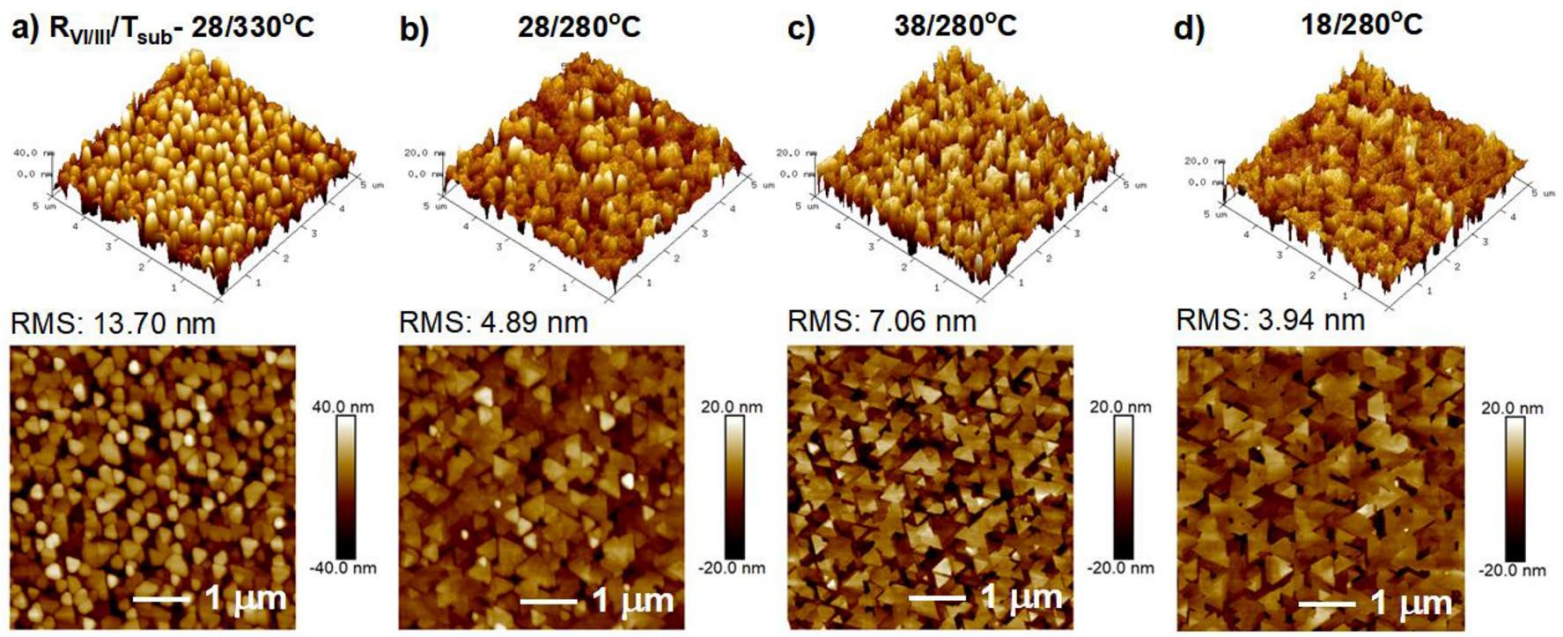
5 × 5 µm^2^ AFM scans of β-In_2_Se_3_ layers grown at R_VI/III_/T_sub_ of (**a**) 28/330 °C, (**b**) 28/280 °C, (**c**) 38/280 °C and (**d**) 18/280 °C epitaxial conditions on HT/LT β-Ga_2_O_3_/c-Sapphire heterostructures.

The epitaxy of the chalcogenide material in a typical solid source UHV-MBE system is employed at a high chalcogen-to-metal flux ratio due to increased volatility and lower sticking coefficient of chalcogen atoms at the growth surface^34^. Owing to this, we maintained Se-rich conditions for the In_2_Se_3_ epitaxy in this work by setting the Se/In flux ratio (R_VI/III_) larger than ~ 15^8^. Figure 4a,b shows the RHEED patterns of the as-grown HT/LT β-Ga_2_O_3_/c-Sapphire before the growth of In_2_Se_3_. When the growth was maintained at T_sub_ of 480 °C (R_VI/III_ ~ 28), the RHEED patterns remained similar to β-Ga_2_O_3_ for the whole growth, as seen in Fig. 4c,d. This indicates that the epitaxy of In_2_Se_3_ layers didn’t occur at these conditions, which might be caused by the kinetic limitations (rate of adsorption and desorption of adatoms) or nucleation barriers^35^ that may not be favorable at 480 °C, leading to hindered growth. Further decreasing the T_sub_ ~ 330 °C, the transition in the RHEED patterns was observed along both the azimuthal directions, as shown in Fig. 4e,f, indicating the change in the crystal structure from monoclinic β-Ga_2_O_3_ to rhombohedral β-In_2_Se_3_ structure. In addition, the streak spacing ratio between the a-a/m-m planes was measured to be ~ $\sqrt{3}$, representing the six-fold symmetry of In_2_Se_3_ layers. Despite the 3D surface morphology of the β-Ga_2_O_3_ film, the In_2_Se_3_ layers were successfully grown on it. This could be a consequence of quasi-vdWs epitaxy being independent of the surface lattice conditions of the underlying layer^36^. The In_2_Se_3_ layers grown on β-Ga_2_O_3_ film followed the in-plane epitaxial relationship of $[11\overline{2 }0]$ β-In_2_Se_3_ || [010] β-Ga_2_O_3_ and $[10\overline{1 }0]$ β-In_2_Se_3_ || [102] β-Ga_2_O_3_. The spotty pattern observed in this condition may have originated from the 3D growth of In_2_Se_3_ layers. Gradually, the RHEED patterns became streakier upon further reducing the T_sub_ ~ 280 °C, indicating improved lateral growth, as shown in Fig. 4g,h. Following this, the In_2_Se_3_ layers were grown with varied R_VI/III_ of 38 and 18 at a T_sub_ of 280 °C. The RHEED patterns became broader with slight spots for the sample grown at an increased flux ratio of 38, Fig. 4i,j, suggesting the declined surface quality. Among the whole series, a clear and streakier pattern was observed for the entire epitaxy when grown at R_VI/III_ ~ 18 (T_sub_ ~ 280 °C), suggesting the improved surface of the In_2_Se_3_ layers, as shown in Fig. 4k,l. Furthermore, the in-plane reciprocal streak spacing along $[10\overline{1 }0]$ β-In_2_Se_3_ with the respective [102] β-Ga_2_O_3_ has yielded the real space ‘a’ lattice constant of β-In_2_Se_3_, ${b}_{{I}_{\left[10\overline{1 }0\right]}}$= 2π/${b}_{{I}_{[10\overline{1 }0]}}^{*}$ Å ~ 4.027 Å as shown Fig. 4m. The surface morphologies of In_2_Se_3_ layers grown on 3D β-Ga_2_O_3_ films at varying epitaxial conditions are shown in Fig. 5 from the 5 × 5 µm^2^ AFM scans. At a T_sub_ of 330 °C (R_VI/III_ ~ 28), we can observe a high density of smaller triangles (~ 250 nm) with a pronounced vertical stacking, resulting in a 3D surface morphology of In_2_Se_3_ (RMS ~ 13.70 nm) which is in correspondence with the observation of spotty RHEED patterns. Further reducing the T_sub_ ~ 280 °C, the density of triangular domains is reduced by exhibiting an improved lateral growth (RMS ~ 4.89 nm). However, increasing the R_VI/III_ to ~ 38 by maintaining the T_sub_ ~ 280 °C resulted in an increment in the density of triangular domains with reduced size. It might be caused by the excess Se atoms occupying the surface sites, causing limited surface diffusion^37^ and further promoting vertical growth, as evidenced by the enhanced RMS ~ 7.09 nm. In contrast, a smoother surface, comprising 0° and 180°-oriented triangles with improved lateral size ~ 450 nm, was observed when the R_VI/III_ was reduced to ~ 18 (RMS ~ 3.94 nm). The step profile analysis reveals the thickness of the monolayer measured to be ~ 0.95 nm, as shown in Fig. S5, which matches well with other reports from the literature^4^. Therefore, we claim that both the T_sub_ and R_VI/III_ play vital roles in controlling the nucleation density and surface quality of the In_2_Se_3_ layers grown on 3D β-Ga_2_O_3_/c-Sapphire.

**Figure 6:**
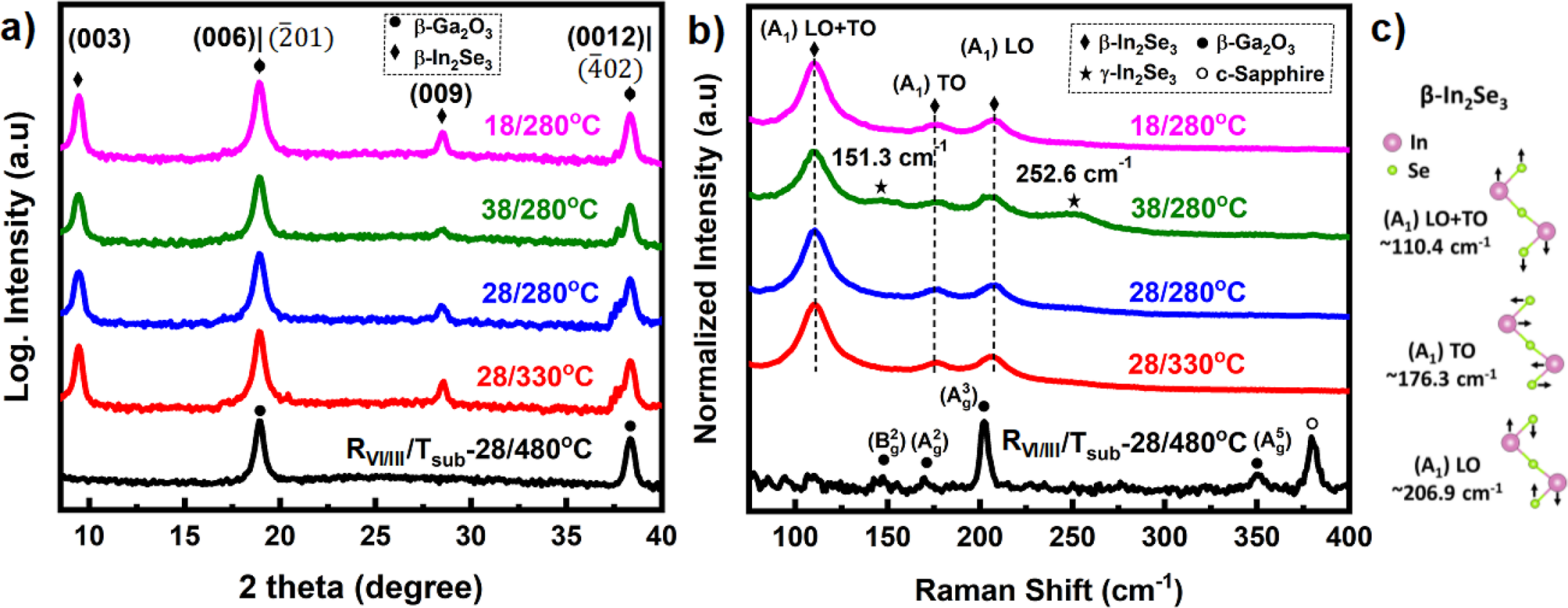
(**a**) XRD 2θ-scans and (**b**) Raman Spectra of β-In_2_Se_3_ layers grown at different R_VI/III_/T_sub_ epitaxial conditions on HT/LT β-Ga_2_O_3_/c-Sapphire heterostructure along with (**c**) active Raman vibrational modes of β-In_2_Se_3_.

On the other hand, it has been a challenging issue to identify and differentiate the commonly obtained crystal phases of In_2_Se_3_, among which the rhombohedral crystal structures of α- and β-In_2_Se_3_ share similar but different space groups (R3m and R$\overline{3 }$m)^5,38^. The primary difference between these two structures lies in the location of In atoms at tetrahedral and octahedral sites covered by the Se packing in α- and β-In_2_Se_3,_ respectively^38^. Subsequently, insisting on a demanding characterization method and prudent analysis to distinguish the respective crystal phases. Figure 6a shows the XRD 2θ-scans of In_2_Se_3_ layers grown on two-stepped β-Ga_2_O_3_/c-Sapphire at different epitaxial conditions. The sample grown at T_sub_ ~ 480 °C (R_VI/III_ ~ 28) exhibits only the diffraction peaks related to β-Ga_2_O_3_, indicating the absence of In_2_Se_3_ epitaxy which agrees well with the observed RHEED patterns. On the other hand, at all the other epitaxial conditions, apart from the diffraction peaks of β-Ga_2_O_3_, we can observe two distinct additional peaks, ~ 9.4° and ~ 28.5° corresponding to (003) and (009) diffraction planes of rhombohedral β-In_2_Se_3_ crystal structure (ICDD Card No. 35–1056, space group R$\overline{3 }$m). Furthermore, no additional peaks are present in the 2θ-scans, confirming the epitaxial growth of single phase β-In_2_Se_3_ layers on 3D two-stepped Ga_2_O_3_ films. However, the peak overlapping at ~ 18.9° and ~ 38.3° originated from the diffraction signals of (006) and (0012) planes of β-In_2_Se_3_ and $(\overline{2 }01)$ and $(\overline{4 }02)$ planes of β-Ga_2_O_3_, respectively, make it difficult to validate the pristine properties of β-Ga_2_O_3_ underneath layers after β-In_2_Se_3_ deposition. Hence, selected 2θ-XRD peak analysis, as well as a direct growth of β-In_2_Se_3_ on c-sapphire, have been performed to support this validation, as shown in Fig. S6(a,b). Among all the samples, the lowest FWHM of (003) and (009) 2θ-diffraction peaks were observed to be ~ 0.29° and ~ 0.35° for the sample grown at T_sub_ ~ 280 °C and R_VI/III_ ~ 18.

Moreover, detailed information is further essential to classify the grown layers. As mentioned earlier, Raman spectroscopy is a robust and non-destructive technique used to characterize the samples with different phases based on the molecular fingerprints obtained from various active phonon modes^1^. Figure 6b shows the Raman spectra of In_2_Se_3_ layers grown at different epitaxial conditions on β-Ga_2_O_3_ films. Here as well, the epitaxy performed at T_sub_ ~ 480 °C (R_VI/III_ ~ 28) shows only peaks related to β-Ga_2_O_3_ film. All the other samples exhibited three clear peaks observed ~ 110.4 cm^−1^, ~ 176.3 cm^−1^, and ~ 206.9 cm^−1^ attributed to A_1_(LO + TO), A_1_(TO), and A_1_(LO) phonon modes, respectively, as shown in Fig. 6c, which are characteristics of β-In_2_Se_3_, that are similar to the previously reported results^1,2,39^. A similar peak overlapping was observed between the pronounced A_g_^3^ mode from the β-Ga_2_O_3_ and the A_1_(LO) mode of β-In_2_Se_3,_ as shown in Fig. S6(c,d). The active vibrational modes exhibited by β-In_2_Se_3_ with regard to the similarly structured α-In_2_Se_3_ are validated upon comparing the typical Raman peaks of α-In_2_Se_3_ as summarized by Liu et al.^39^. However, the sample grown at T_sub_ of 280 °C under the R_VI/III_ ~ 38 exhibited two additional Raman modes ~ 151.3 cm^−1^ and ~ 252.6 cm^−1^, along with the respective β-In_2_Se_3_ modes. These peaks are characteristics of γ-In_2_Se_3_, with the former phonon mode corresponding to the zone center vibration and the latter to the excess contribution of Se atoms’ linkage to the Se-Se bond due to the high Se flux used in this series^40,41^. This indicates that, at these epitaxial conditions, the growth leads to the co-existence of β-In_2_Se_3_ and γ-In_2_Se_3_ with the dominance in the former phase. The existence of additional 3D γ-In_2_Se_3_ may cause predominantly vertical growth, resulting in a rougher surface, as evident from the surface morphology characterization mentioned above.

**Figure 7:**
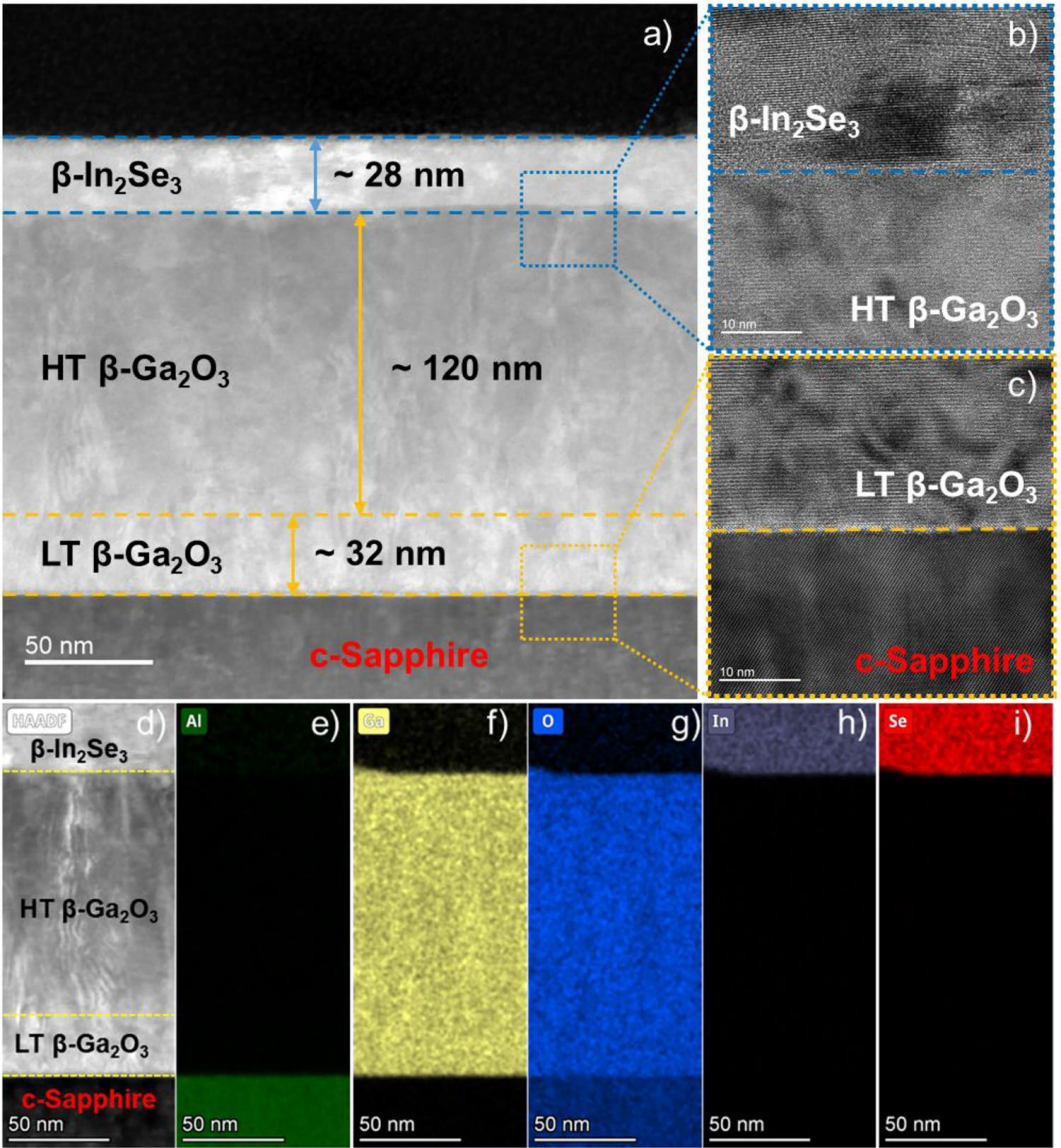
(**a**) Low magnification STEM-HAADF cross-sectional view of the β-In_2_Se_3_/β-Ga_2_O_3_ heterostructure grown on c-Sapphire; (**b**,**c**) represent the high-magnification TEM images of the interfaces between β-In_2_Se_3_/HT β-Ga_2_O_3_ and LT β-Ga_2_O_3_/c-Sapphire, respectively. (**d**) Low magnification STEM-HAADF cross-sectional view of β-In_2_Se_3_/β-Ga_2_O_3_/c-Sapphire heterostructure grown on c-Sapphire showing the corresponding elemental mappings (**e**–**i**) of Al, Ga, O, In, and Se atoms in their respective films.

Furthermore, Fig. 7a shows the STEM high-angle annular dark-field (HAADF) cross-sectional view of the β-In_2_Se_3_/β-Ga_2_O_3_ heterostructure grown on c-Sapphire. The thicknesses of the LT-, HT β-Ga_2_O_3,_ and β-In_2_Se_3_ films are determined to be ~ 32 nm, ~ 120 nm, and ~ 28 nm, respectively, with the corresponding growth rates of ~ 0.53, ~ 1.0, and ~ 0.47 nm/min. Figure 7b,c provides a detailed visualization of the interfaces between the LT β-Ga_2_O_3_/c-Sapphire and β-In_2_Se_3_/HT β-Ga_2_O_3_ heterostructures. Regardless of the relatively rough surface of the two-stepped β-Ga_2_O_3_ film that may result in a non-abrupt 2D/3D interface, the layered structure of β-In_2_Se_3_ is clearly observed, attributed to the quasi-van der Waals epitaxy. The detailed elemental mappings of the entire β-In_2_Se_3_/β-Ga_2_O_3_ heterostructure grown on c-Sapphire are shown in Fig. 7d–i, which reveal an abrupt transition and uniform distribution of respective elements within the specific layers. These results evidently support the objective of the present work on realizing the epitaxial growth of β-In_2_Se_3_ on β-Ga_2_O_3_, which shows the potential scope to study the mixed dimensional heterostructures for future device applications using MBE technique.

So far, we have successfully achieved the epitaxy of the single phase 2D β-In_2_Se_3_ layers on 3D two-stepped β-Ga_2_O_3_/c-Sapphire. In the present series, the better structural and surface quality of β-In_2_Se_3_ layers was obtained when grown at R_VI/III_ and T_sub_ of ~ 18 and ~ 280 °C, respectively. Finally, such a mixed dimensional (2D β-In_2_Se_3_/3D β-Ga_2_O_3_) heterostructure can avail the benefits offered by both materials, specifically in the optoelectronic field, with its absorption edges extending from Near-IR (~ 1.43 eV)^1^ to deep UV regions (~ 5.04 eV), and can be used as a dual-band photodetector. Also, the epi-grown In_2_Se_3_ and Ga_2_O_3_, being intrinsically n-type, can form a heterostructure exhibiting an nN isotype heterojunction, forming 2DEG upon bandgap engineering. Hence, studying the band alignment of this heterostructure can unveil new opportunities in the (opto-) power electronic field.

In conclusion, we successfully realized 2D β-In_2_Se_3_/3D β-Ga_2_O_3_ heterostructures on c-Sapphire substrates using the PA-MBE technique. A two-stepped Ga_2_O_3_ growth was employed to improve the crystalline quality of the film, as indicated by the XRD 2θ-scans and Raman Spectra. For the first time, the in-plane ‘b’ lattice constant of β-Ga_2_O_3_ (~ 3.038Å) grown on c-Sapphire was determined using in-situ RHEED patterns. In the next stage, the 2D β-In_2_Se_3_ layers were successfully grown on 3D β-Ga_2_O_3_ films resulting from quasi-vdWs epitaxy. The In_2_Se_3_ layers followed an in-plane epitaxial relationship of $[11\overline{2 }0]$ β-In_2_Se_3_ || [010] β-Ga_2_O_3_ and $[10\overline{1 }0]$ β-In_2_Se_3_ || [102] β-Ga_2_O_3_ with the in-plane lattice constant of β-In_2_Se_3_ determined to be ~ 4.027Å. The single phase β-In_2_Se_3_ layers with improved structural and surface quality were achieved when growth was maintained at R_VI/III_ ~ 18 and T_sub_ ~ 280 °C on β-Ga_2_O_3_/c-Sapphire. The (S)TEM microstructural and detailed elemental analysis has clearly indicated the successful realization of 2D β-In_2_Se_3_/3D β-Ga_2_O_3_ heterostructure on c-Sapphire, completely in-situ using PA-MBE. Such an epitaxial realization of 2D layers on 3D films can enhance the potential of mixed-dimensional heterostructures by increasing the scalability and reducing the possibility of contamination compared to other transfer methods. The realized β-In_2_Se_3_/β-Ga_2_O_3_ heterostructure with its optical bandgap energies (E_g_) ~ 1.43 eV (Near-IR)^1^ and ~ 5.04 eV (Deep UV), respectively, has potential applications in the field of optoelectronics.

## Experimental methods

The epitaxy of Ga_2_O_3_ and In_2_Se_3_ thin films was performed by the SVT associates PA-MBE system at a background pressure of ~ 2 × 10^–10^ torr, using the Knudsen cells with high purity Gallium (7N), Indium (6N), and Selenium (6N) sources. The active oxygen species for the Ga_2_O_3_ growth was supplied by a Radio-frequency (RF) Plasma source. Firstly, a two-stepped Ga_2_O_3_ film was grown on the c-Sapphire substrate at LT and HT conditions. The Ga cell beam equivalent pressure (BEP) and T_sub_ for the epitaxy under LT and HT conditions were 2 × 10^–8^ torr and 450 °C, 6 × 10^–8^ torr and 700 °C, respectively. The oxygen plasma source was maintained at an RF power of 300W with a flow rate of 1.0 sccm for the two-stepped growth. The growth times of LT- and HT-Ga_2_O_3_ films were one and two hours, respectively. After the epitaxy of two-stepped Ga_2_O_3_ thin film, a series of In_2_Se_3_ layers were grown at different T_sub_ (480°–280 °C) and at varying Se/In BEP flux ratios (R_VI/III_) (18–38) by maintaining a constant R_VI/III_ of 28 and T_sub_ of 280 °C respectively, for 1 h. The Se and In BEPs used in the present series range between 5.0–6.75 × 10^–7^ and 1.8–2.85 × 10^–8^ torr, respectively. The in-situ surface reconstructions of the films during the growth were monitored by RHEED operated at an electron beam energy of 12 keV. The surface morphology of the as-grown films was investigated using the atomic force microscope (AFM, Bruker Dimension Icon). The crystal quality and phase characterization of the films were determined by X-ray diffraction (XRD, Bruker New D8 Discover) 2θ-scans using Cu-Kα radiation (λ = 1.54056 Å) and Raman Spectrum using a LabRam iHR550 HORIBA spectrometer under 532 nm laser excitation. The microstructural and interfacial analysis at atomic resolutions was determined using the (Scanning) Transmission Electron Microscope (S)TEM (FEI Talos F200X, ThermoFisher Scientific), operated at 200 kV. The optical transmittance spectra were obtained using a JASCO V-780 UV–Vis-NIR Spectrophotometer. The in-plane and out-of-plane crystallographic views of β-Ga_2_O_3_ on c-Sapphire along the growth direction were visualized using the ball and stick model by VESTA Software version 3.5.7.

### Supplementary Information

Supplementary Information.

## Data Availability

The data used during this study are available from the corresponding author, W.-C.C upon reasonable request.
