# Supplementary material for: Structural and surface characterizations of 2D β-In2Se3/3D β-Ga2O3 heterostructures grown on c-Sapphire substrates by molecular beam epitaxy
Source: Sci Rep. 2024 Mar 1;14:5146. doi: 10.1038/s41598-024-55830-y (PMC10907755; doi:10.1038/s41598-024-55830-y)
Supplement: Supplementary file 1 — Supplementary Information. [file 41598_2024_55830_MOESM1_ESM.docx]

SUPPLEMENTARY INFORMATION for

**Structural and Surface Characterizations of 2D β-In_2_Se_3_/3D β-Ga_2_O_3_ Heterostructures Grown on c-Sapphire Substrates by Molecular Beam Epitaxy**

Umeshwar Reddy Nallasani^1^, Ssu-Kuan Wu^1^, Nhu Quynh Diep^1^, Yen-Yu Lin^1^, Hua-Chiang Wen^1^, Wu-Ching Chou^1,*^ and Chin-Hau Chia^2^

*^1^Department of Electrophysics, College of Science, National Yang Ming Chiao Tung University, 1001 University Road, Hsinchu 300093, Taiwan, R.O.C.*

*^2^Department of Applied Physics, National University of Kaohsiung, 700 University Road, Kaohsiung 81148, Taiwan, R.O.C.*

**^*^*Corresponding author:*** [*wcchou957@nycu.edu.tw*](mailto:wcchou957@nycu.edu.tw)


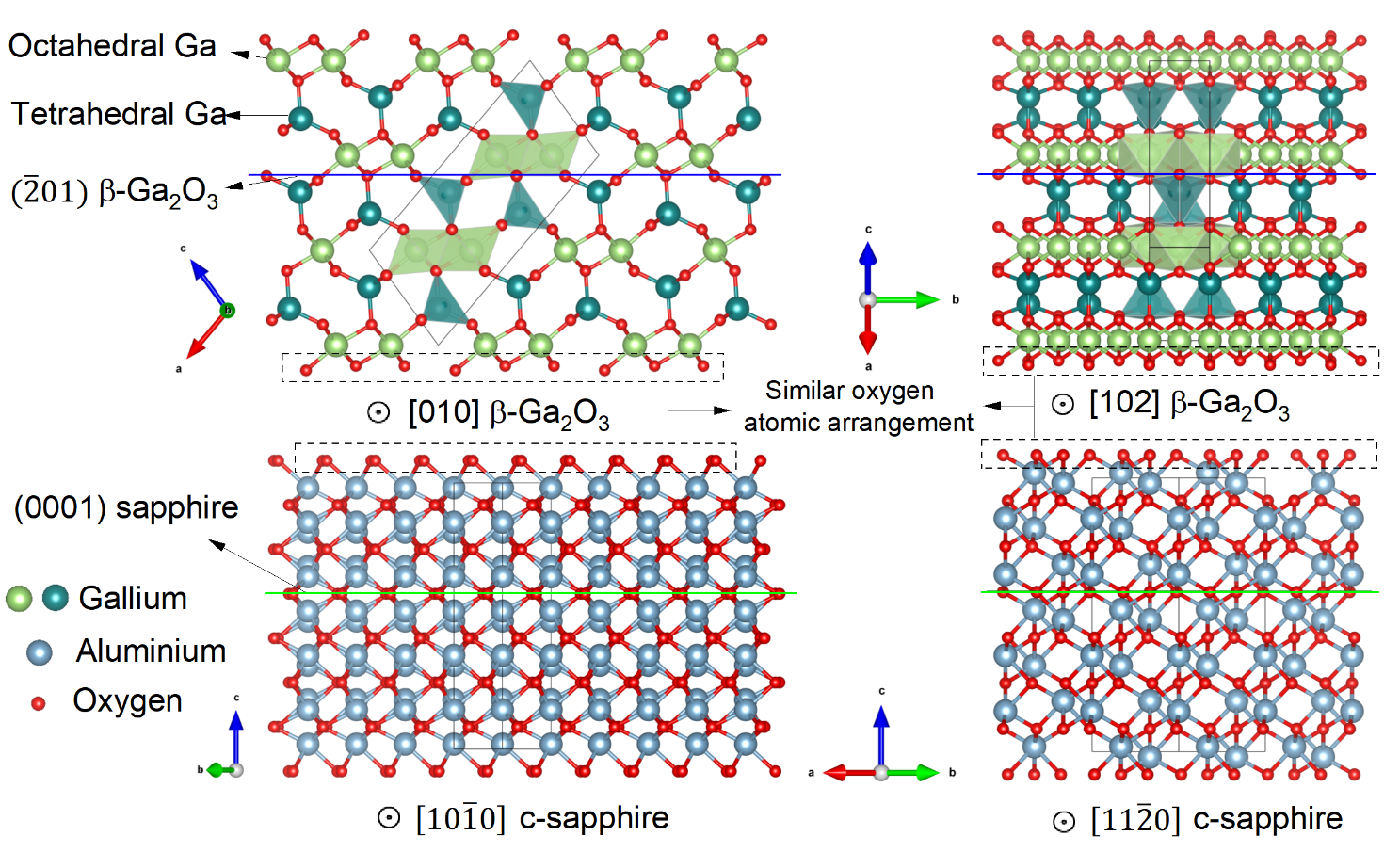


**Figure S1**. The out-of-plane directional views of β-Ga_2_O_3_ along [010] and [102], c-Sapphire along $[10\bar{1}0]$ and $[11\bar{2}0]$ visualized using the ball and stick model by VESTA software^1^.

Figure S1 shows the ball and stick model of out-of-plane directional views of β-Ga_2_O_3_ ([010] and [1 02]) and c-Sapphire ($[10\bar{1}0]$ and $[11\bar{2}0]$) that is repeated for every 60^o^ of rotation. The polar $(\bar{2}01)$ plane of β-Ga_2_O_3_ consists of oxygen or gallium atoms. We can observe a similar array of oxygen atoms within both layers in their respective directions. In β-Ga_2_O_3,_ the gallium atoms are arranged in a tetrahedral and octahedral pattern within the monoclinic structure, as shown in two colors.


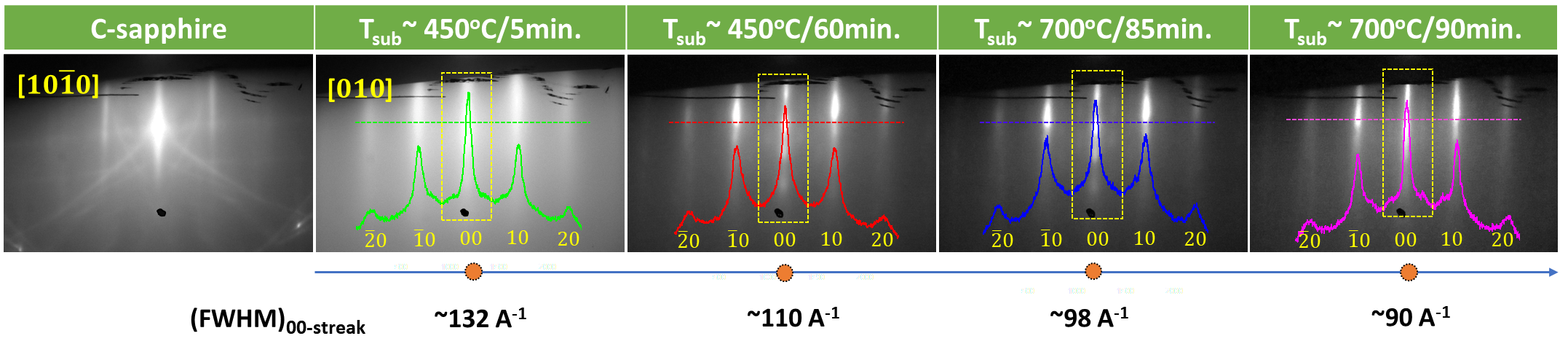


**Figure S2.** Temporal evolution of RHEED patterns and intensity profiles along [010] Ga_2_O_3_ at different T_sub_. The dotted line indicates the position (same for all) where the intensity profiles are extracted. The yellow dotted box indicates the FWHM of 00 peaks extracted from different time intervals and T_sub_.

Figure S2 shows the evolution of RHEED patterns from the two-stepped β-Ga_2_O_3_ film grown on c-sapphire taken along [010] azimuthal direction at various stages of the deposition. In the first stage of the growth, the FWHM of the 00-streak line of LT β-Ga_2_O_3_/sapphire exhibited significant narrowing from ~132 Å^-1^ at 5 min. to ~110 Å^-1^ as finishing its growth (60 min.) at the substrate temperature (T_sub_) ~450^o^C. This may suggest the lattice mismatch compensation and uniform nucleation in the LT-Ga_2_O_3_ film (as reinforced by the smooth surface from the AFM scan in Fig. 2b of the main text), ready for the HT/LT-Ga_2_O_3_ homoepitaxy in the next step. During the transitioning for HT film growth, the T_sub_ was increased to 700^o^C at 10^o^C/min., while opening the oxygen plasma source. Interestingly, we observed the FWHM of 00-streak further decreased to ~98 Å^-1^ after reaching the T_sub_ of 700^o^C, suggesting the recrystallization of the film, and this particularly provides a moderately decent surface for HT film growth than c-sapphire. As a result, only a few minutes after starting the deposition of HT-Ga_2_O_3_, the FWHM of 00-streak keeps reducing to ~90 Å^-1^, suggesting an improvement in film quality.


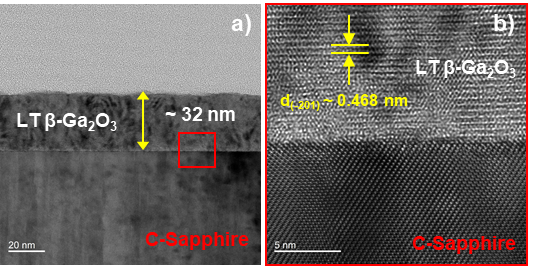


**Figure S3.** (a) Low magnification TEM cross-sectional view of LT β-Ga_2_O_3_/c-Sapphire with the thickness of LT β-Ga_2_O_3_ film measured to be ~ 32nm and (b) a high magnification view showing the d-spacing of $(\bar{2}01)$ planes of β-Ga_2_O_3_ ~ 0.468nm.


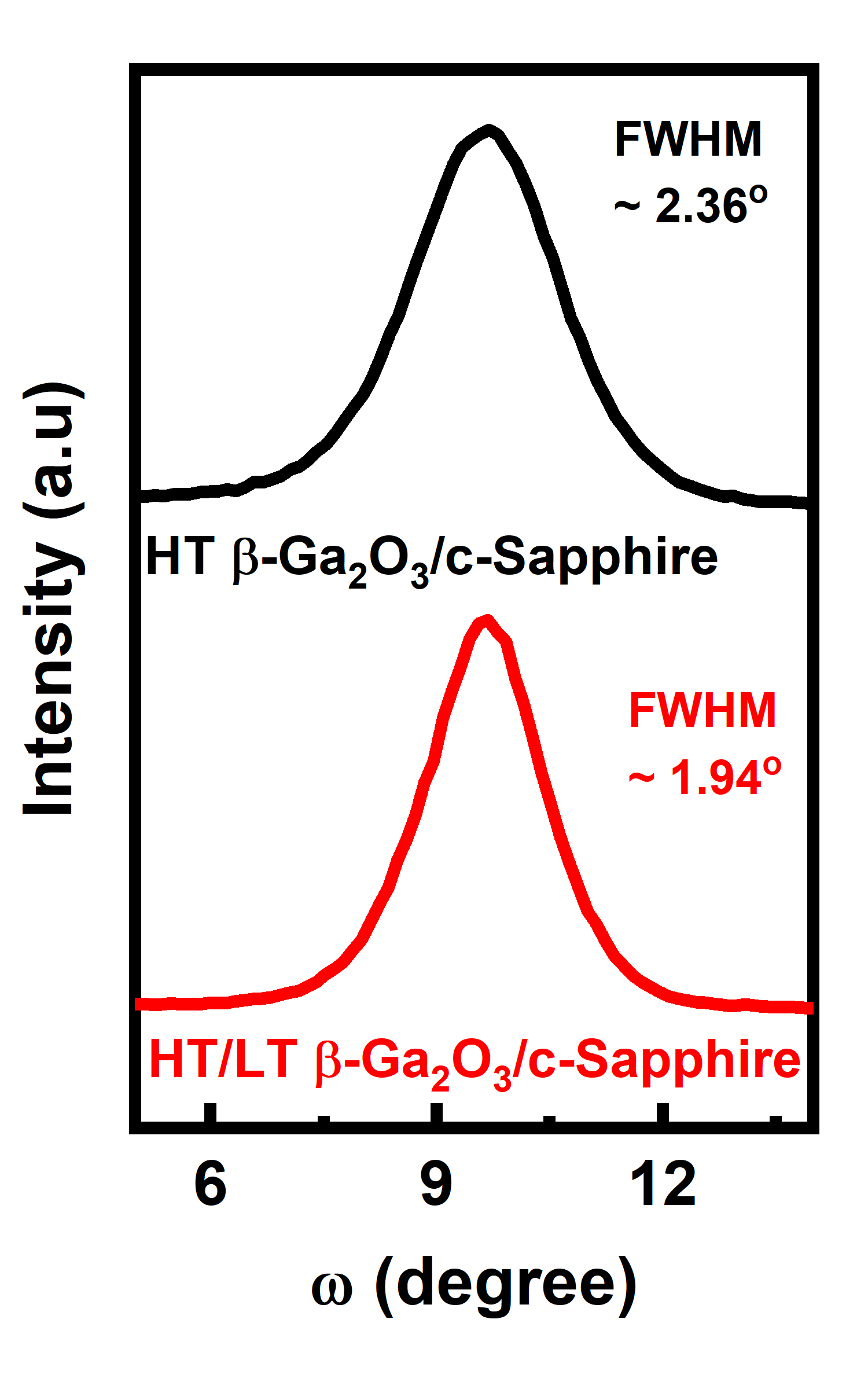


**Figure S4.** X-ray rocking curves (XRCs) for the $(\bar{2}01)$ plane of the β-Ga_2_O_3_ films grown without and with a LT β-Ga_2_O_3_ nucleation film on c-Sapphire.

Figure S4 illustrates the XRCs for the $(\bar{2}01)$ plane of β-Ga_2_O_3_ grown on c-Sapphire without and with LT β-Ga_2_O_3_ nucleation film. The FWHM of the β-Ga_2_O_3_ film notably decreased from 2.36^o^ (without LT β-Ga_2_O_3_ film) to 1.94^o^ (with a ~ 32nm LT β-Ga_2_O_3_ film), indicating an enhancement in the crystalline quality of β-Ga_2_O_3_ with the insertion of the LT film.

**
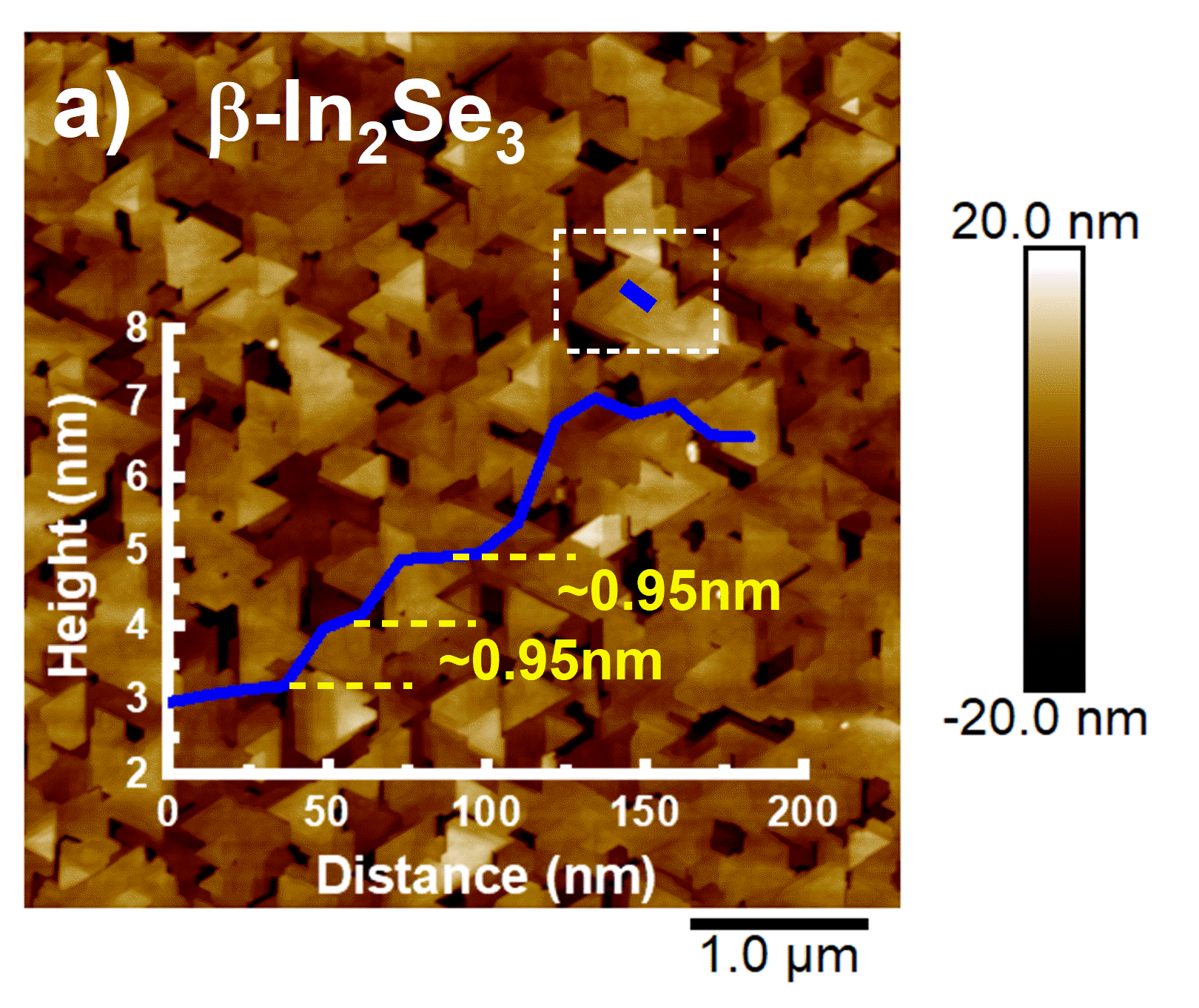
**

**Figure S5**. 5×5 µm^2^ AFM scan of β-In_2_Se_3_ grown on the β-Ga_2_O_3_/c-Sapphire sample at R_VI/III_/T_sub_ of 18/280^o^C, with the thickness of monolayer measured to be ~ 0.95nm.


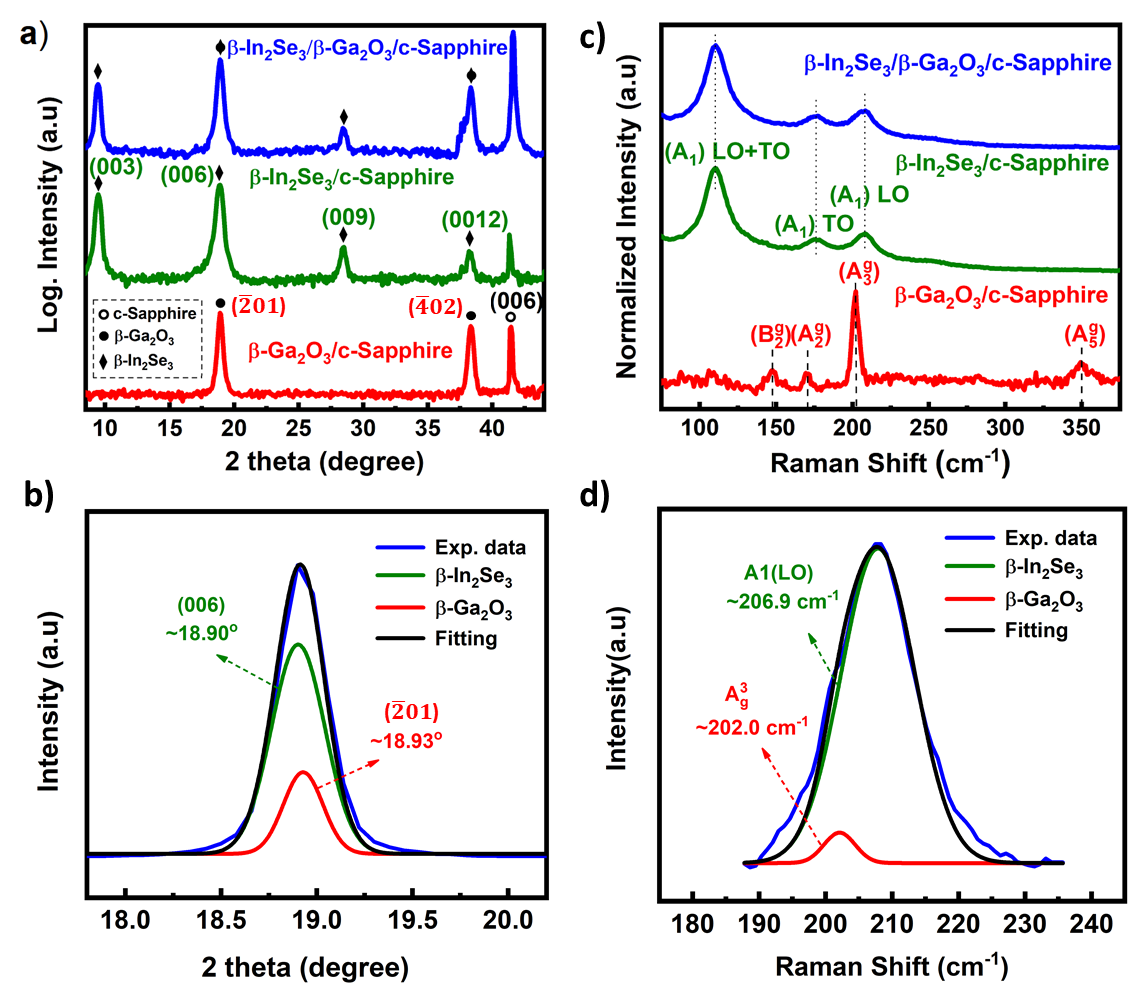


**Figure S6**. (a) XRD 2θ-scans of β-Ga_2_O_3_ and β-In_2_Se_3_ grown on c-Sapphire and β-In_2_Se_3_/β-Ga_2_O_3_/c-Sapphire samples. (b) Multiple-peak fitting of 2θ diffraction peak observed at ~18.9^o^. (c) Raman spectrum of β-Ga_2_O_3_ and β-In_2_Se_3_ grown on c-Sapphire and β-In_2_Se_3_/β-Ga_2_O_3_/c-Sapphire samples and (d) Multiple-peak fitting of Raman vibrational mode observed at ~207 cm^-1^.

Figure S6(a) shows the XRD 2θ-scans of β-Ga_2_O_3_/c-Sapphire, β-In_2_Se_3_/c-Sapphire, and β-In_2_Se_3_/β-Ga_2_O_3_/c-Sapphire, we can observe the alternative positions of (00*l*) β-In_2_Se_3_ diffraction planes overlaps with every peak of $(\bar{2}01)$ β-Ga_2_O_3_. This correspondence indicates that the lattice d-spacing of these films is remarkably consistent at specific diffraction planes. To validate this, the high-intensity 2θ-XRD peak located at ~ 18.9^o^ is selected for peak analysis using the Gaussian fitting function, as shown in Fig. S6(b). The result unveils separate peaks corresponding to (006) and $(\bar{2}01)$ diffraction peaks of β-In_2_Se_3_ (~18.90^o^) and β-Ga_2_O_3_ (~18.93^o^), respectively. Moreover, it is found that the FWHM value of the $(\bar{2}01)$ β-Ga_2_O_3_ peak in the presence of top In_2_Se_3_ layer is comparable to that of the HT/LT-Ga_2_O_3_ film (FWHM ~0.28^o^), suggesting the pristine properties of Ga_2_O_3_ layer after In_2_Se_3_ growth. Similarly, Fig. S6(c) shows the Raman Spectra of β-Ga_2_O_3_/c-Sapphire, β-In_2_Se_3_/c-Sapphire, and β-In_2_Se_3_/β-Ga_2_O_3_/c-Sapphire. The pronounced A_g_^3^ active mode of β-Ga_2_O_3_ overlaps with the (A_1_) LO mode of β-In_2_Se_3_ as shown clearly in Fig. S6(d) by the peak splitting of Raman vibrational mode observed ~ 207 cm^-1^ in the spectrum.

Notably, irrespective of the available surface growth front, i.e., c-Sapphire or β-Ga_2_O_3_/c-Sapphire, the In_2_Se_3_ layers maintained β-phase when grown under the same epitaxial conditions, similarly observed by X. Zhang et al. when In_2_Se_3_ was grown on c-Sapphire and Si (111) substrates^2^.

**References**

1 Momma, K. & Izumi, F. VESTA: a three-dimensional visualization system for electronic and structural analysis. *Journal of Applied crystallography* **41**, 653-658 (2008).

2 Zhang, X. *et al.* Epitaxial growth of few-layer β-In_2_Se_3_ thin films by metalorganic chemical vapor deposition. *Journal of Crystal Growth* **533**, 125471 (2020).
